# A comparative study of spike protein of SARS-CoV-2 and its variant Omicron (B.1.1.529) on some immune characteristics

**DOI:** 10.1038/s41598-022-21690-7

**Published:** 2022-10-12

**Authors:** Ximeng Li, Wenjing Li, Zhuangzhuang Liu, Yuan Kang, Xiaoyu Zhang, Zhenlu Xu, Yuan Gao, Yun Qi

**Affiliations:** Research Center for Pharmacology and Toxicology, Institute of Medicinal Plant Development, Chinese Academy of Medical Science and Peking Union Medical College, 151, North Ma Lian Wa Road, Haidian District, Beijing, 100193 People’s Republic of China

**Keywords:** Inflammation, Innate immunity

## Abstract

The emergence of Omicron variant raises great concerns because of its rapid transmissibility and its numerous mutations in spike protein (S-protein). S-protein can act as a pathogen-associated molecular pattern and complement activator as well as antigen. We compared some immune characteristics of trimer S-proteins for wild type (WT-S) and B.1.1.529 Omicron (Omicron-S) to investigate whether the mutations have affected its pathogenicity and antigenic shift. The results indicated that WT-S and Omicron-S directly activated nuclear factor-κB (NF-κB) and induced the release of pro-inflammatory cytokines in macrophages, but the actions of Omicron-S were weaker. These inflammatory reactions could be abrogated by a Toll-like receptor 4 antagonist TAK-242. Two S-proteins failed to induce the production of antiviral molecular interferon-β. In contrast to pro-inflammatory effects, the ability of two S-proteins to activate complement was comparable. We also compared the binding ability of two S-proteins to a high-titer anti-WT-receptor-binding domain antibody. The data showed that WT-S strongly bound to this antibody, while Omicron-S was completely off-target. Collectively, the mutations of Omicron have a great impact on the pro-inflammatory ability and epitopes of S-protein, but little effect on its ability to activate complement. Addressing these issues can be helpful for more adequate understanding of the pathogenicity of Omicron and the vaccine breakthrough infection.

## Introduction

The world has been suffering from the pandemic of COVID-19, caused by SARS-CoV-2, for more than 2 years. Similar to other coronaviruses, spike protein (S protein), a trimer on the surface of SARS-CoV-2, is the virus “key” to infect host. As a crucial antigen for developing antibodies and vaccines, S-protein is composed of two functional subunits-S1 and S2. The former can bind with the host cell receptor angiotensin-converting enzyme 2 (ACE2) via its subdomain the receptor binding domain (RBD), while the latter is responsible for the fusion of viral and host cell membranes. The mutations in SARS-CoV-2 have led to significant changes in variants transmission and resistance mechanisms against the host immune system. In contrast to 2–3 mutations in RBD of other variants of concern, Omicron contains 15 missense substitutions in RBD, which is not only the vital binding site for the entry of SARS-CoV-2, but also the key target of neutralizing antibodies generated by infections or vaccination^1–3^.

Indeed, Omicron has exhibited a stronger vaccine-breakthrough capability than any other previous variants^1^. Several studies have demonstrated that Omicron evades the majority of existing SARS-CoV-2 antibodies^4–8^. In a high-throughput screening study, 210/247 (> 85%) of the tested monoclonal antibodies (mAbs) failed to bind Omicron^4^. But luckily, it is also because of these mutations that Omicron shows less efficient replication and weaker fusion activity than previous variants^9–11^. Moreover, reports from South Africa have consistently noted that the rate of hospitalization as a result of Omicron infections is lower, strongly suggesting that Omicron may cause milder disease^12^.

Toll-like receptors (TLRs), which play key roles in COVID-19^13^, can identify a series of pathogen-associated molecular patterns (PAMPs) and induce a robust inflammatory response through myeloid differentiation factor 88 (MyD88)- or TIR-domain-containing adapter-inducing interferon-β (TRIF)-dependent pathway. Not surprisingly, as an enveloped positive-stranded RNA virus, SARS-CoV-2 can provoke TLR7/8-mediated inflammatory responses^14^. Unusually, the SARS-CoV-2 S-protein alone, without the rest of the viral components, can act as a PAMP to trigger inflammation^15–19^. For example, S-protein can directly induce macrophage inflammation via TLR2 or TLR4 signaling^17,18^. Such features raise the question of whether the mutations of S-protein have affected its pathogenicity and antigenic shift. In this study, we chose two intact trimer-S-proteins, the prototypes in SARS-CoV-2, for wild type (WT) and Omicron (B.1.1.529), respectively, and compared their immunology-related characteristics, including the ability to induce inflammation or activate complement, and the affinity for anti-WT-RBD antibody. Addressing these issues can be helpful for more adequate understanding of the pathogenicity of Omicron and the vaccine breakthrough infections.

## Materials and methods

### Reagents

Trimer WT S-protein (WT-S; M.W. 409.8 KD; 1.83 mg protein/mL; Cat^#^DRA122), trimer B.1.1.529 Omicron S-protein (Omicron-S; M.W. 415.2 KD; 0.35 mg protein/mL; Cat^#^DRA193) and WT RBD protein (Cat^#^DRA32), derived from mammalian expression system (human hek 293 cells), were prepared by Novoprotein Co. (Suzhou, Jiangsu, China) (Supplementary Note). Chromogenic Endotoxin Quant Kit was from Pierce (Rockford, IL, USA). TAK-242, C29 and Pam3CSK4 (Pam) were from MedChemExpress (Monmouth Junction, NJ, USA). Commercial ELISA kits for mouse tumor necrosis factor-α (TNF-α; Cat^#^430904, sensitivity 4 pg/mL), interleukin-6 (IL-6; Cat^#^431304, sensitivity 2 pg/mL), monocyte chemotactic protein-1 (MCP-1; Cat^#^432704, sensitivity 30 pg/mL), interferon (IFN)-β (Cat^#^439407, sensitivity 1.9 pg/mL), and human MCP-1 (Cat^#^438804, sensitivity 3.9 pg/mL) were from Biolegend Co. (San Diego, CA, USA). Normal human serum complement and mouse anti-human C5b-9 antibody (aE-11) were from Quidel Corporation (San Diego, CA, USA) and Santa Cruz Biotechnology, Inc. (Dallas, TX, USA), respectively. Mouse mAb isotyping test kit was from RoChe Ltd. (Mannheim, Germany). LPS was from Sigma-Aldrich (St. Louis, MO, USA). The nuclear factor-κB (NF-κB)-TA-luc plasmid and the firefly luciferase reporter gene assay kit was from Beyotime Institute of Biotechnology (Haimen, Jiangsu, China). Horseradish peroxidase (HRP)-conjugated anti-mouse IgG secondary antibody was from ABclonal Biotech Co. (Wuhan, Hubei, China).

### Cells and animals

Mouse RAW264.7 macrophages and human THP-1 cells were obtained from ATCC and Institute of Basic Medical Sciences of Chinese Academy of Medical Sciences (CAMS), respectively. They were cultured in a humidified incubator with 5.0% CO_2_ at 37 °C. BALB/c mice (female, 18–20 g) used for preparing mAb were from Vital River Experimental Animal Services (Beijing, China) and housed in a vivarium under the standard conditions of temperature and humidity and with a 12 h light/dark cycle.

### Ethics statement

In this study, all methods were performed in accordance with relevant guidelines and regulations including the Guide for the Care and Use of Laboratory Animals^20^. The reporting in the manuscript follows the recommendations in the ARRIVE guidelines^21^. Moreover, anesthetic drug (isoflurane) and euthanasia were used to reduce animals suffering during experimental procedures according to American Veterinary Medical Association (AVMA) Guidelines for the Euthanasia of Animals^22^. All animal care and experimental protocols and procedures had been approved by the Committee for Care and Welfare of Laboratory Animals in Institute of Medicinal Plant Development of Chinese Academy of Medical Sciences & Peking Union Medical College.

### Measurement of supernatant cytokines

RAW264.7 macrophages were treated with trimer WT-S or Omicron-S at different concentrations. The cells in negative control group were treated with vehicle. Twenty-four hours later, supernatant cytokines (e.g., TNF-α, MCP-1, IL-6 and IFN-β) were determined using their commercial ELISA kits. Briefly, capture antibody solution (100 μL/well) was added and the plates were incubated at 4 °C overnight. Wash the unbound antibody with wash buffer for 4 times. Assay diluent (200 μL/well) was added and the plates were incubated at 25 °C for 1 h with shaking. Wash plate 4 times, add diluted standards or samples and incubate the plate at 25 °C for 2 h with shaking. After washing plate 4 times, diluted detection antibody solution (100 μL/well) was added and the plates were incubated at 25 °C for 1 h with shaking. After washing again, diluted avidin-HRP solution (100 μL/well) was added and the plates were incubated at 25 °C for 30 min with shaking. Before developing chromogenic reaction, wash plate 5 times and add TMB substrate solution (100 μL/well). The plates were incubated in the dark for 15–30 min. Stop solution (100 μL/well) was added and the absorbance was read at 450 nm within 15 min. All standards and samples were run in triplicate.

For the antagonist experiments, the cells were treated with S-protein in the presence of TAK-242 (1 μM) or C29 (40 μM). Twenty-four hours later, the levels of TNF-α, MCP-1, IL-6 and IFN-β in supernatant were determined. The cells treated with vehicle (0 group) were used as the negative control.

### Luciferase assay

RAW264.7 macrophages stably transfected with NF-κB-TA-luc plasmid were treated with test substances (10 ng/mL LPS or 300 ng/mL Pam or trimer S-protein) with or without antagonist (TAK-242 or C29) for 4 h. The cells were lysed, then the luciferase activity in the lysate was measured using the luciferase assay system according to the manufacturer’s instruction. Briefly, the cells were rinsed with PBS for 3 times and completely lysed by lysis buffer (200 μL/well). The cell lysate was centrifugated at 13,000 × g for 5 min at 4 °C and the supernatant was collected. The supernatant (50 µL) was added to a white 96-well plate followed by 50 µL of luciferase assay reagent. Then the chemiluminescence was measured immediately by MicroBeta2 microplate counter (PerkinElmer, MA, USA).

### Preparation of mAb against WT SARS-CoV-2 RBD

The mAb against WT SARS-CoV-2 RBD was prepared as we previously described^23^.

### Measurements of complement serum function and antibody affinity^24,25^

For complement serum functional assay, the 96-well microtiter plates coated with trimer WT-S or Omicron-S (0–48 nM, 100 μL/well) were blocked by 1% bovine serum albumin (BSA) and then incubated at 37 °C for 2 h. Unbound S-proteins were washed by PBST, and 16% normal human serum complement was added. The reactions continued at 37 °C for 1 h and terminated by washing by PBST. Then, 100 μL of mouse anti-human C5b-9 IgG2b antibody was added and the plate was incubated at 37 °C for 1 h. Unbound antibodies were washed, and HRP-conjugated goat anti-mouse IgG was added and the plate was incubated at 37 °C for 30 min. After washing five times, add 100 μL of TMB substrate to each well and incubate for 10 min in the dark. The reaction was terminated by 2 M H_2_SO_4_ and the optical density (OD) values at 450_ nm_ were determined. For antibody affinity assay, the plates coated with trimer WT-S or Omicron-S (2.4 nM, 100 μL/well) were incubated with purified RBD mAb at different concentrations at 37 °C for 1 h. Then, HRP-conjugated goat anti-mouse IgG antibody was added and TMB chromogenic reactions were developed as described above. The antibody affinity was negatively correlated to the antibody concentration corresponding to 50% of the maximum OD value.

### Data analysis

The data were reported as the mean ± SD from a representative experiment. All of the experiments reported in this work were repeated at least three times with the same pattern of results. All data were analyzed by GraphPad Prism 8.0 using one-way ANOVA followed by the *Tukey posttest*^26^. A student’s *t*-test was used when only two groups were compared. *P* < 0.05 was considered significant.

## Results

### WT-S exerts stronger pro-inflammatory ability compared with Omicron-S

To rule out the possibility of false-positive results caused by endotoxin contamination, we first determined the endotoxin levels of two S-proteins using Pierce™ Chromogenic Endotoxin Quant Kit. The data showed that the endotoxin concentrations of the stock solutions of WT-S (1.83 mg protein/mL) and Omicron-S (0.35 mg protein/mL) were 1.43 ng/mL and 0.187 ng/mL, respectively. In our cell system, the highest final concentration of S-proteins was 2.4 nM (≈ 1 μg/mL), which translated to the highest final concentrations of endotoxin derived from WT-S and Omicron-S to be 0.78 pg/mL and 0.53 pg/mL, respectively. In comparison, the lowest endotoxin concentration that could concurrently induce three cytokines’ release was 500 pg/mL (Supplementary Fig. S1). Therefore, the effects of the two S-proteins observed in this study were irrelevant to endotoxin.

**Figure 1:**
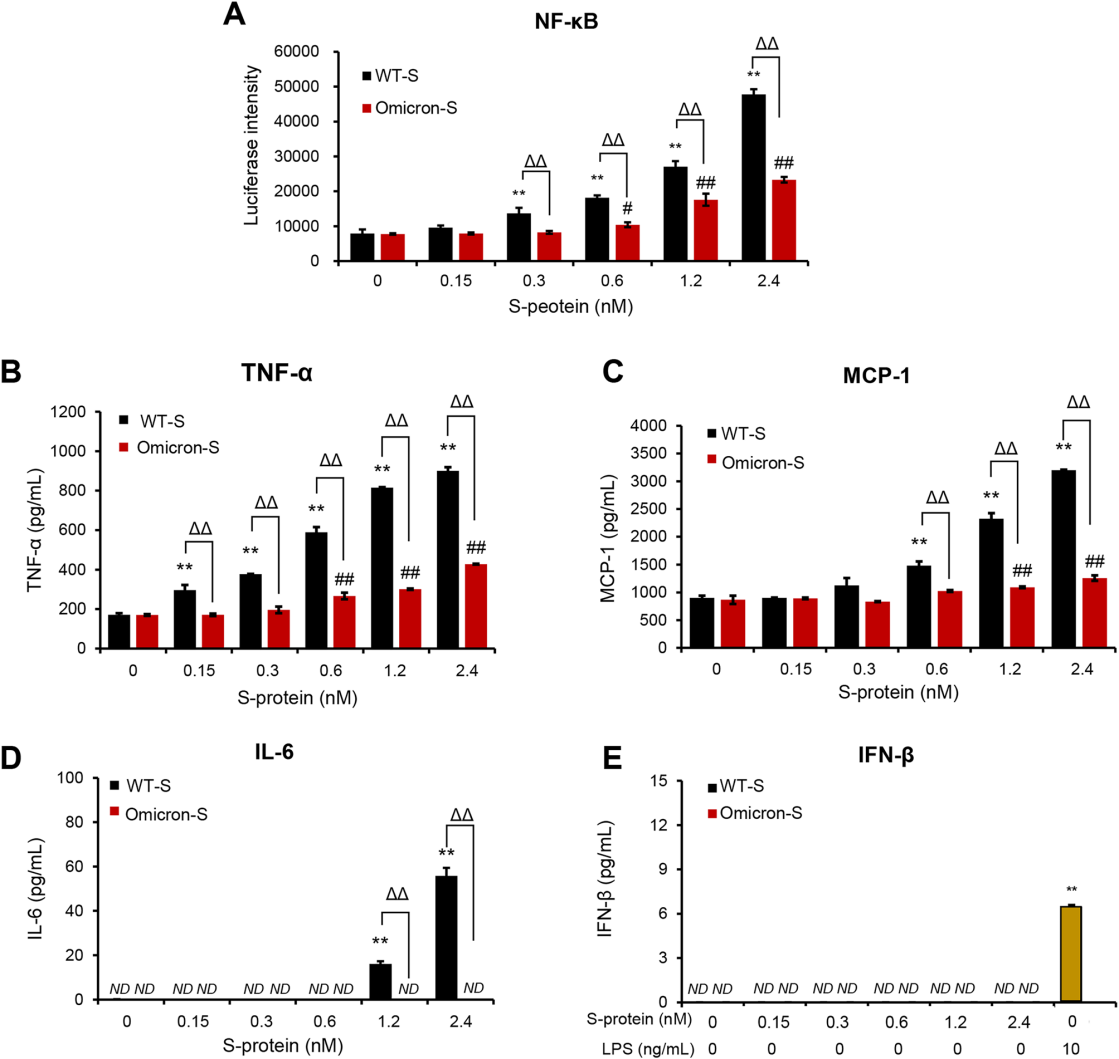
Comparison of the effects of trimer WT-S and Omicron-S on inflammatory responses in RAW264.7 macrophages. (**A**) Effects of trimer WT-S and Omicron-S on NF-κB activity. The cells stably transfected with NF-κB-TA-luc plasmid were stimulated with trimer WT-S or Omicron-S at the indicated concentrations. Four hours later, the luciferase activities were detected. (**B–E**) Effects of trimer WT-S and Omicron-S on supernatant TNF-α, MCP-1, IL-6 and IFN-β in RAW264.7 macrophages. Cells were treated with trimer WT-S or Omicron-S at the indicated concentrations. Twenty-four hours later, supernatant TNF-α, MCP-1, IL-6 and IFN-β were determined. LPS was used as a positive control for INF-β. Mean ± SD (n = 3). **P < 0.01 *vs*. 0 nM of WT-S (negative control); ^#^*P* < 0.05 and ^##^*P* < 0.01 *vs*. 0 nM of Omicron-S (negative control); ^ΔΔ^*P* < 0.01. *ND*, not detected.

Subsequently, we compared the pro-inflammatory ability of WT-S and Omicron-S in RAW264.7 macrophages. The effects of two S-proteins on NF-κB activity were first determined. As shown in Fig. 1A, 0.3 nM of WT-S and 0.6 nM of Omicron-S could trigger NF-κB activation, and their actions increased with the higher concentrations. At the same concentration, WT-S exerted stronger action compared with Omicron-S. Next, we compared their effects on supernatant cytokines. The results showed that WT-S elevated supernatant TNF-α, MCP-1 and IL-6 in a concentration-dependent manner, while Omicron-S only increased TNF-α and MCP-1 at higher concentrations, showing weaker pro-inflammatory activity (Fig. 1B-D). Notably, both WT-S and Omicron-S could not induce the production of IFN-β, an antiviral cytokine, although in parallel experiment LPS could do (Fig. 1E).

### Antagonist for TLR4 but not TLR2 can block the pro-inflammatory effects of WT-S and Omicron-S

**Figure 2:**
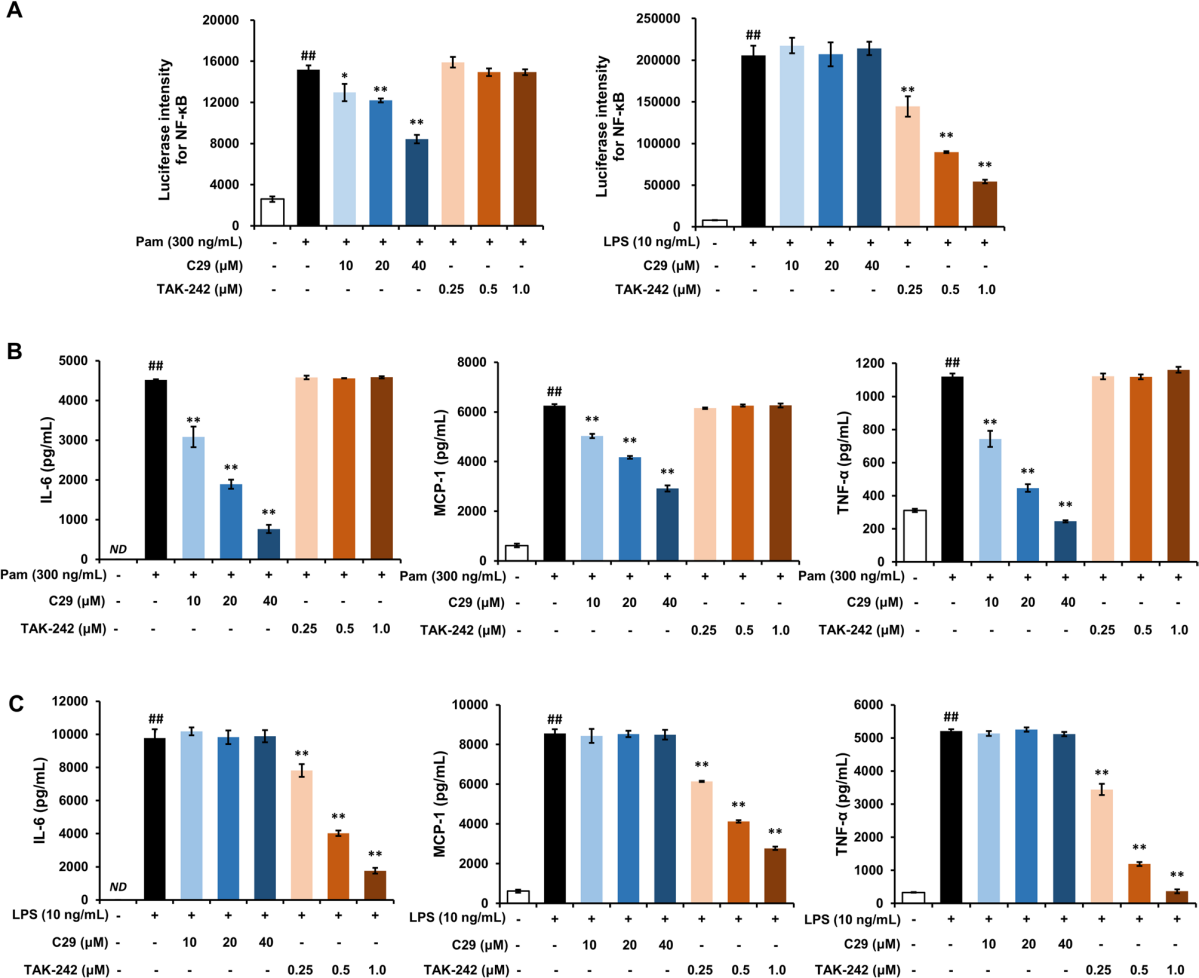
Effects of C29 and TAK-242 on Pam- or LPS-induced pro-inflammatory reactions in RAW264.7 macrophages. (**A**) Effects of C29 and TAK-242 on Pam- or LPS-induced NF-κB activation in RAW264.7 macrophages. (**B**) Effects of C29 and TAK-242 on Pam-elevated supernatant IL-6, MCP-1 and TNF-α in RAW264.7 macrophages. (**C**) Effects of C29 and TAK-242 on LPS-elevated supernatant IL-6, MCP-1 and TNF-α in RAW264.7 macrophages. Mean ± SD (n = 3). ^##^*P* < 0.01 *vs*. negative control; *P < 0.05 and **P < 0.01 *vs*. Pam or LPS alone. *ND*, not detected.

**Figure 3:**
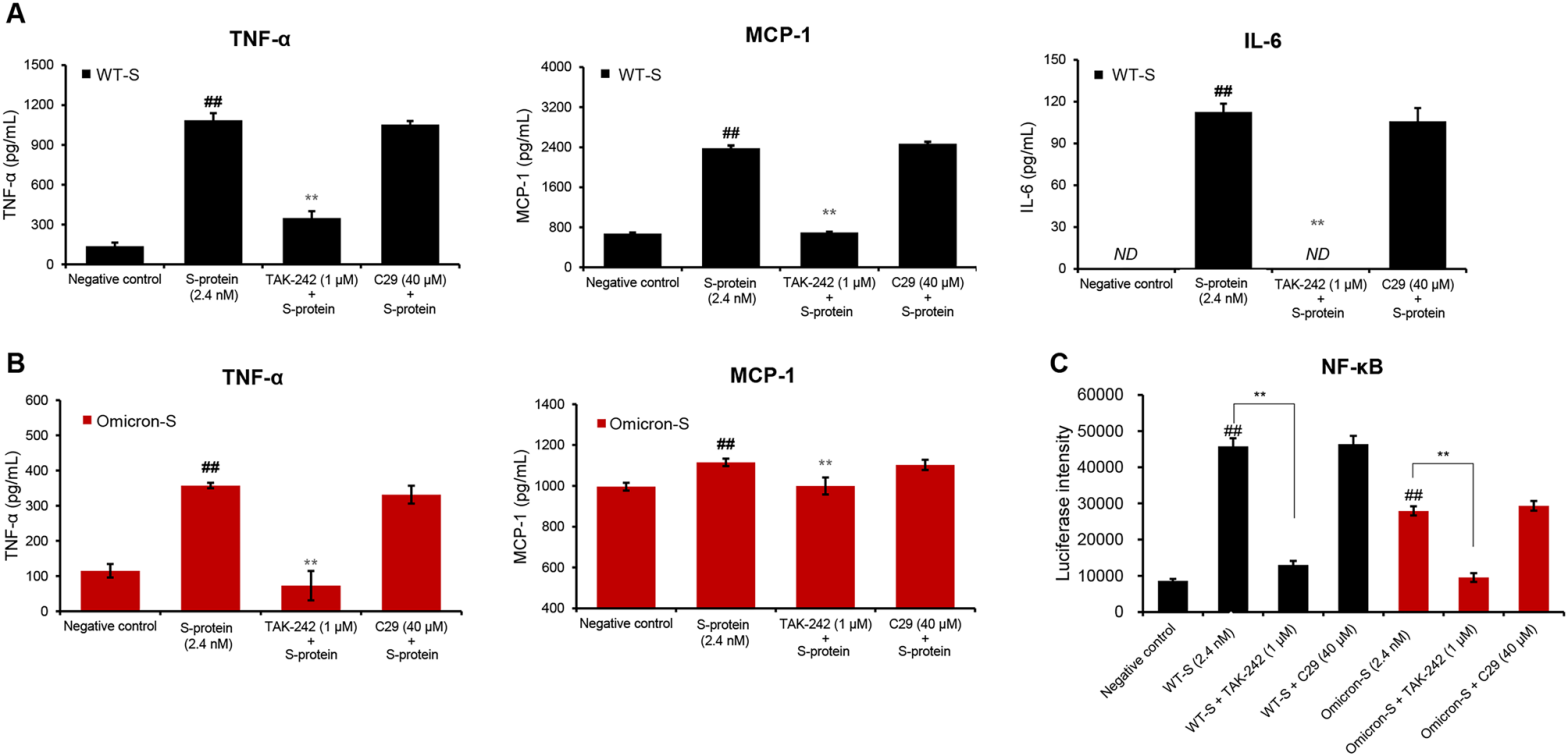
Effects of TAK-242 and C29 on the pro-inflammatory reactions induced by two trimer S-proteins. (**A–B**) Effects of TAK-242 and C29 on supernatant TNF-α, MCP-1 and IL-6 induced by WT-S (**A**) or Omicron-S (**B**) in RAW264.7 macrophages. The cells were treated with trimer WT-S or Omicron-S (2.4 nM) in the presence of TAK-242 (1 μM) or C29 (40 μM). Twenty-four hours later, supernatant TNF-α, MCP-1 and IL-6 were determined. *ND*, not detected. (**C**) Effects of TAK-242 and C29 on trimer WT-S- or Omicron-S-induced NF-κB activation. RAW264.7 cells stably transfected with NF-κB-TA-luc plasmid were treated with C29 (40 μM) or TAK-242 (1 μM) and then stimulated by trimer WT-S or Omicron-S (2.4 nM). Four hours later, the luciferase activities were detected. Mean ± SD (n = 3). ^##^*P* < 0.01 *vs*. negative control; **P < 0.01 *vs*. WT-S or Omicron-S alone.

As a PAMP, SARS-CoV-2 S-protein possesses its pattern recognition receptor (PRR) to induce inflammation. Two incompatible PRRs, TLR2 and TLR4, were proposed by different research teams^17,18^. In our study, two S-proteins failed to induce IFN-β production (Fig. 1E). Therefore, their PRR was more likely to be TLR2 because TRIF activation was not involved in TLR2 signaling. To confirm this speculation, TLR2 antagonist C29^27^ and TLR4 antagonist TAK-242^28^ were used for blocking ligand-induced interaction of TLRs with their adaptor molecules. Firstly, we ascertained their effective concentrations in LPS (a TLR4 ligand)- or Pam (a TLR1/2 ligand)-stimulated RAW264.7 macrophages. As a result, 60 μM of C29 exerted an off-target action on LPS (TLR4 ligand)-induced inflammatory responses (Supplementary Fig. S2). Thus, 40 μM of C29 and 1 μM of TAK-242 were chosen in the subsequent antergic experiments (Fig. 2). It was observed that TAK-242, instead of C29, nearly completely counteracted the productions of pro-inflammatory cytokines induced by WT-S and Omicron-S (Fig. 3A–B). Moreover, two S-proteins-induced NF-κB activation was also inhibited by TAK-242 (Fig. 3C). Consistent effect could also be detected on human THP-1 cells (Supplementary Fig. S3). These results demonstrated that it was through activating TLR4, rather than TLR2, for two S-proteins to trigger inflammation.

### Comparison of the ability of WT-S and Omicron-S to activate complement

**Figure 4:**
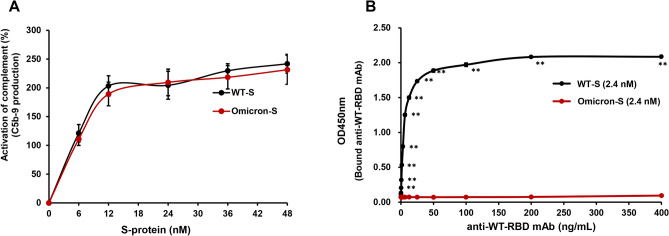
Comparison of the ability of trimer WT-S and Omicron-S to activate complement and bind to anti-WT-RBD mAb. (**A**) Comparison of the ability of trimer WT-S and Omicron-S to activate complement. Normal human serum complement was added into the plate coated with trimer WT-S or Omicron-S at different concentrations (0–48 nM). After 1 h incubation, complement activation was terminated by washing the plate four times. The produced membrane attack complex C5b-9 was combined with a mouse anti-human C5b-9 IgG2b antibody which further combined with an HRP-conjugated goat anti-mouse IgG antibody. The chromogenic reaction was developed by adding TMB substrate and then terminated by 2 M H_2_SO_4_. OD values at 450_ nm_, which were positively correlated with the C5b-9 production, were determined. Activation percentage of complement was calculated relative to the negative control. Mean ± SD (n = 3). (**B**) Comparison of the binding ability of two trimer S-proteins to anti-WT-RBD mAb. The purified anti-WT-RBD mAb at different concentrations was added into the plate coated with 2.4 nM of trimer WT-S or Omicron-S. After 1 h incubation, the unbound mAb was washed away by PBST and the bound mAb could be detected by HRP-conjugated goat anti-mouse IgG. The chromogenic reaction was developed by adding TMB substrate and then terminated by 2 M H_2_SO_4_. OD values at 450_ nm_, which were positively correlated with the binding ability (affinity) of S-protein to anti-WT-RBD mAb, were determined. Mean ± SD (n = 3). **P < 0.01 *vs*. Omicron-S.

During SARS-CoV-2 infection, complement activation can lead to local and systemic damage in some of severe COVID-19^29,30^. Moreover, SARS-CoV-2 S-protein can directly activate complement via non-classical pathways^31,32^. We further compared the ability of two S-proteins to activate complement. As shown in Fig. 4A, both WT-S and Omicron-S indeed activated complement at the indicated concentrations. However, unlike pro-inflammatory responses (Fig. 1A–D), complement activation triggered by both of them was comparable (*P* > 0.1).

### Comparison of the affinity of anti-SARS-CoV-2 RBD mAb for WT-S and Omicron-S

Omicron is of great concern to the world due to its more than 30 mutations that may have an impact on transmissibility and immune evasion^33^. More importantly, 15 mutations are clustered in the S-protein RBD which is not only the vital binding site to the host receptor ACE2 for the entry of virus, but also the key target of neutralizing antibodies after infection or vaccination^3^. We chose RBD of WT-S as an antigen and prepared its high-titer mAb (IgG2b). Subsequently, the affinity of two trimer S-proteins for this RBD mAb was compared. The data showed that WT-S strongly bound to this mAb; but in comparison, the affinity of Omicron-S for this mAb was completely lost, rather than reduced (Fig. 4B), indicating that the original antigenic epitopes of RBD have been thoroughly mutated in Omicron.

## Discussion

The innate immune inflammatory response can be initiated by the recognition of PAMPs by PRRs, such as TLRs, NOD-like receptors (NLRs), and RIG-I like receptors (RLRs). Activated PRRs involve multiple signaling adapters to activate transcription factors which regulate their respective target genes. Usually, viral single-stranded RNA can provoke inflammatory responses via its PRRs (e.g., TLR7/8)^14^. It was recently reported that the S-protein of SARS-CoV-2 itself also can act as a PAMP to activate TLRs^17,18^. Through the comparative studies, we found that both WT-S and Omicron-S indeed induced NF-κB activation (Fig. 1A) and the release of pro-inflammatory cytokines (Fig. 1B–D; Supplementary Fig. S3). Moreover, their effects could be nearly abrogated by the TLR4 antagonist TAK-242 (Fig. 3; Supplementary Fig. S3), suggesting that they were through activating MyD88-dependent pathway to trigger pro-inflammatory responses.

TRIF is another adaptor molecule that initiates TRIF-dependent pathway for TLR4 to produce type I IFNs. A usual TLR4 ligand (e.g., LPS) can activate these two pathways in the meantime. As a novel TLR4 ligand, two S-proteins unexpectedly failed to activate TRIF signaling (Fig. 1E). The unusual case once made us doubt the accuracy of previous antagonist experiment, because the ineffective type I IFN response is a hallmark of TLR2 signaling and TLR2 was also reported to be a PRR for S-protein^17^. After repeated experiments, we confirmed that only the antagonist for TLR4 was able to block the inflammation triggered by S-protein. From the virus perspective, activating TLR4 without promoting the production of IFN-β is obviously more conducive to its persistence and replication. Obviously, S-protein can initiate an unbalanced immune response, namely a weak production of IFN-β and a hypersecretion of pro-inflammatory cytokines, which also is the characteristic of SARS-CoV-2-infected immune cells^34,35^.

We found that although two S-proteins could promote the productions of TLR4-mediated pro-inflammatory cytokines, the effect of Omicron-S was weaker (Fig. 1B–D; Supplementary Fig. S3). Consistently, the latest research also indicated that Omicron was less pathogenic in rodent models than prior SARS-CoV-2 variants^36^. Clinically, increasing evidence shows that Omicron variant infection was associated with significantly lower odds of moderate or severe/critical disease^37–41^. Therefore, it is worth to further investigate whether the weaker pro-inflammatory ability of S-protein directly contributes to the milder virulence of Omicron.

The complement system also is an ancient and important part of the innate immune arsenal against pathogens. Accumulated evidences indicated that over-activation of the complement system may contribute to the cytokine storm, endothelial inflammation (endotheliitis) and thrombosis in the most severe forms of COVID-19^29,42^. Moreover, S-protein can be directly recognized by lectin or alternative pathway components, thus leading to complement activation^31,32^. Our data showed that the ability of two S-proteins to activate complement was comparable (Fig. 4A) although there were more than 30 mutations in Omicron-S.

Previous studies have shown that most of the mutations that contribute to decreased antibody binding and increased immune escape are located on the RBD^43^. Among 15 mutations in RBD of Omicron-S, 13 of them are predicted to affect the antibody neutralization through either altering the S-protein conformation or changing its surface charge distribution. Besides 4 mutations (E484A, K417N, N501Y, T478K)^2^, other 9 mutations (G339D, G446S, N440K, Q493R, Q498R, S371L, S373P, S375F, Y505H) are peculiar to Omicron^1,44,45^. In fact, Omicron has exerted potent capability for immune evasion in the presence of most therapeutic SARS-CoV-2 mAbs^4–8^, showing that the affinity of Omicron-S for previous SARS-CoV-2 mAb has become weaker. We just prepared a high-titer mAb against the RBD of WT-S. By using this mAb, we can evaluate the impact of RBD mutations on antibody affinity. The subsequent result showed that the mAb completely lost the binding activity against Omicron-S (Fig. 4B), indicating that these mutations can evade the immune response. This process is known as antigenic shift^46^. Coincidentally, Cameroni and colleagues also reported that most mAbs, which recognized RBD epitopes, lost the neutralizing activity against Omicron *in vitro*^46^. In this respect, calling Omicron "the great escape artist" is not an exaggeration^47^.

In summary, the present study firstly compared the ability of two S-proteins to induce inflammation and activate complement, as well as their affinity for WT-RBD mAb. Our findings show that the mutations attenuate the pro-inflammatory ability of S-protein, which may be associated with the milder symptoms caused by Omicron infection. Meanwhile, the ability of Omicron-S to activate complement is not significantly affected. These results provide insight into the complexity of mutations on the immune characteristics of S-protein. Moreover, the binding ability of WT-RBD mAb to Omicron-S was completely lost, suggesting the successful immune escape of Omicron. If our mAb is representative, previously designed neutralizing antibodies or vaccines based on the RBD of WT-S may face great challenges.

## Supplementary Information

Supplementary Information.

## Data Availability

The datasets generated during and/or analyzed during the current study are available from the corresponding author on reasonable request.
