# Supplementary material for: A comparative study of spike protein of SARS-CoV-2 and its variant Omicron (B.1.1.529) on some immune characteristics
Source: Sci Rep. 2022 Oct 12;12:17058. doi: 10.1038/s41598-022-21690-7 (PMC9554390; doi:10.1038/s41598-022-21690-7)
Supplement: Supplementary file 1 — Supplementary Information. [file 41598_2022_21690_MOESM1_ESM.pdf]

Supplementary files

A comparative study of spike protein of SARS-CoV-2 and its variant Omicron (B.1.1.529) on some immune characteristics

Ximeng Li, Wenjing Li, Zhuangzhuang Liu, Yuan Kang, Xiaoyu Zhang, Zhenlu Xu, Yuan Gao✉ and Yun Qi✉

Supplementary Note. The detailed information for two S-proteins

1. Intact trimer-S-proteins for WT SARS-CoV-2 (WT-S)

1.83 mg/mL; Cat#DRA122; Lot#0332078MA002;

https://www.novoprotein.com.cn/Cn/product/inproduct/id/3084/catid/90.html

Description:

Recombinant SARS-CoV-2 S-stable trimer Protein is produced by our Mammalian expression system with a 6His tag at the C-terminus. Proline substitutions and alanine substitutions are introduced to stabilize the trimeric prefusion state of SARS-CoV-2 S protein and abolish the furin cleavage site, respectively. Six proline substitutions and furin cleavage site substitutions are introduced to stabilize the trimeric prefusion state of SARS-CoV-2 S protein and abolish the furin cleavage site, respectively.

Accession: QHD43416.1

2. Intact trimer-S-proteins for Omicron (Omicron-S, B.1.1.529 lineage)

0.35 mg/mL; Cat#DRA193; Lot#0332413MM063;

https://www.novoprotein.com.cn/Cn/product/inproduct/id/3262/catid/90.html

Description:

Recombinant SARS-CoV-2 S-trimer Protein is produced by Mammalian expression system and the target gene encoding Cys15-Gln1208 is expressed with a 6His tag at the C-terminus. A67V, HV69-70del, T95I, G142D, VYY143-145del, N211del, L212I, ins214EPE, G339D, S371L, S373P, S375F, K417N, N440K, G446S, S477N, T478K, E484A, Q493R, G496S, Q498R, N501Y, Y505H, T547K, D614G, H655Y, N679K, P681H, N764K, D796Y, N856K, Q954H, N969K and L981F mutations were identified in the SARS-CoV-2 variant (known as Omicron/B.1.1.529 lineage) which emerged in South Africa. Two proline substitutions and furin cleavage site substitutions are introduced to stabilize the trimeric prefusion state of SARS-CoV-2 S protein and abolish the furin cleavage site, respectively.

Accession: P0DTC2

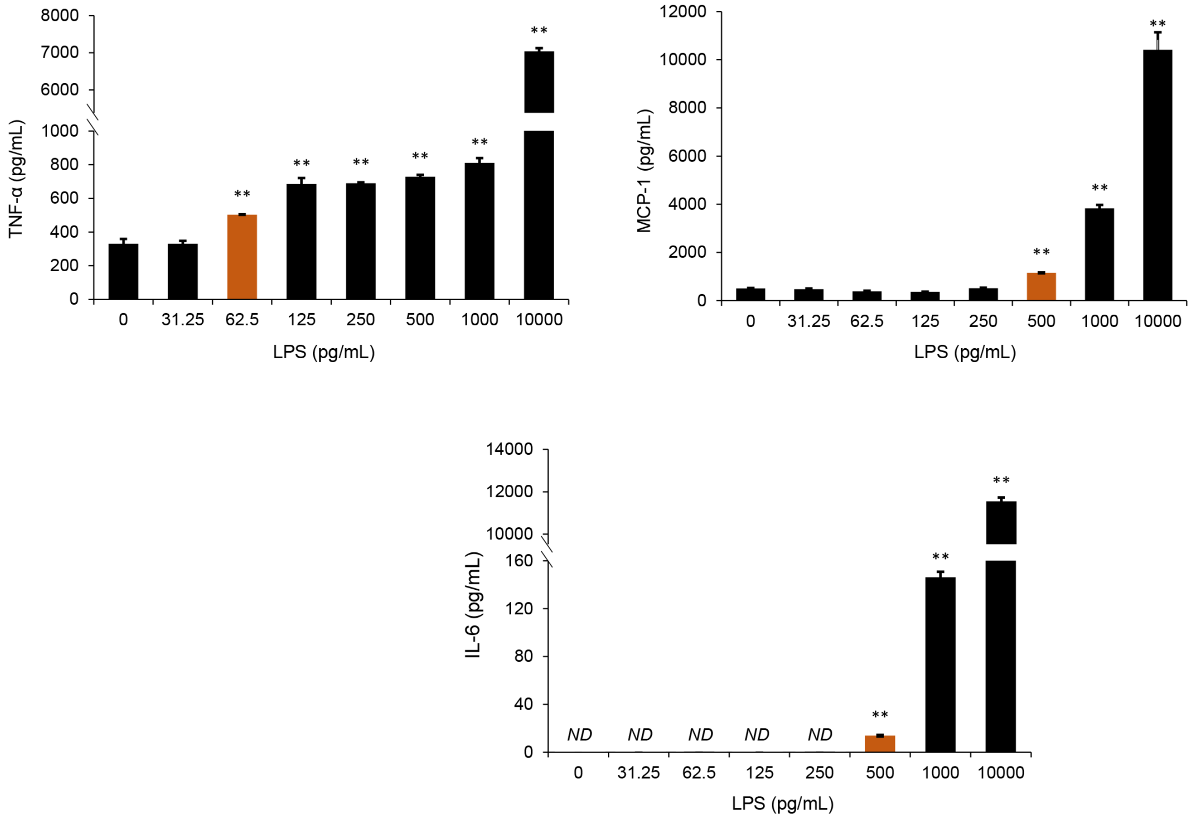

Supplementary Figure S1. Responses of RAW264.7 macrophages to LPS at different concentrations. The cells were treated with LPS at the indicated concentrations. Twenty-four hours later, supernatant TNF-α, MCP-1 and IL-6 were determined using their commercial ELISA kits. Their concentrations were calculated from the standard curves. Mean ± SD (n = 3). ** $P < 0.01$ *vs*. 0 pg/mL of LPS (negative control). *ND*, not detected.

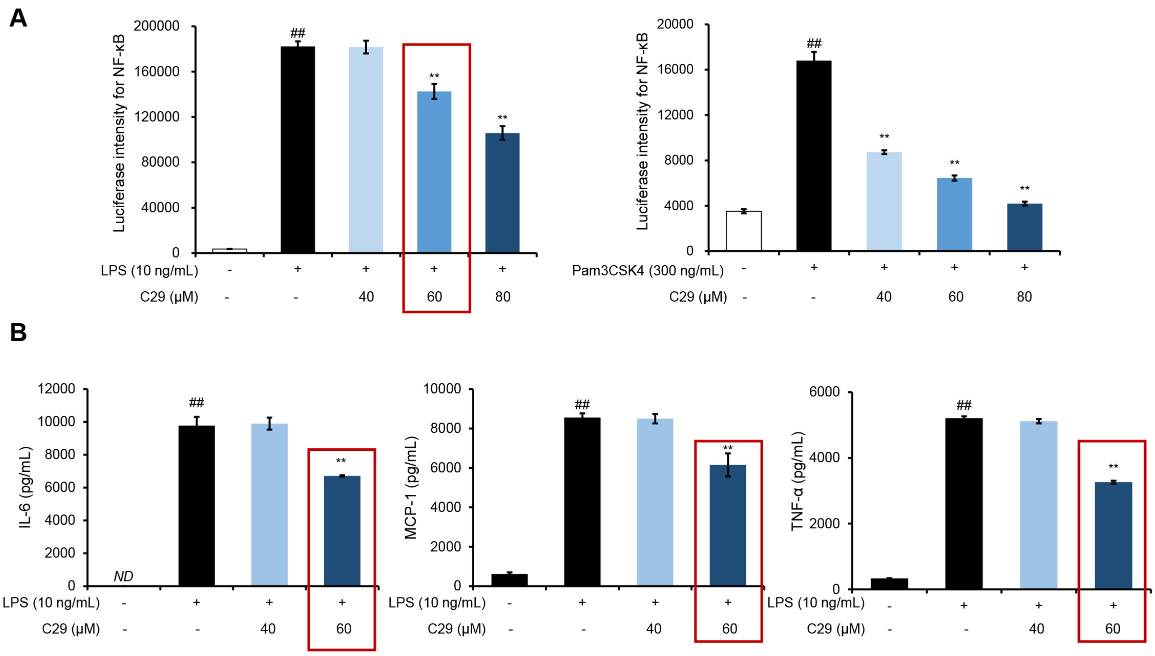

Supplementary Figure S2. C29 (≥ 60 μM) was able to inhibit LPS-induced inflammation. (A) Effects of C29 on LPS- or Pam3CSK4-induced NF-κB activation in RAW264.7 macrophages. The cells transfected with NFκB-TAluc plasmid were stimulated with Pam3CSK4 or LPS in the presence or absence of C29 at indicated concentrations. Four hours later, the luciferase activities were detected by using luciferase assay kit. Mean ± SD (n = 3). ## $P < 0.01$ vs. negative control; ** $P < 0.01$ vs. Pam3CSK4 or LPS alone. (B) Effects of C29 on supernatant TNF-α, MCP-1 and IL-6 induced by LPS in RAW264.7 macrophages. The cells were treated with LPS in the presence of C29 at the indicated concentrations. Twenty-four hours later, supernatant TNF-α, MCP-1 and IL-6 were determined using their commercial ELISA kits. Their concentrations were calculated from the standard curves. Mean ± SD (n = 3). ## $P < 0.01$ vs. negative control; ** $P < 0.01$ vs. LPS alone. *ND*, not detected.

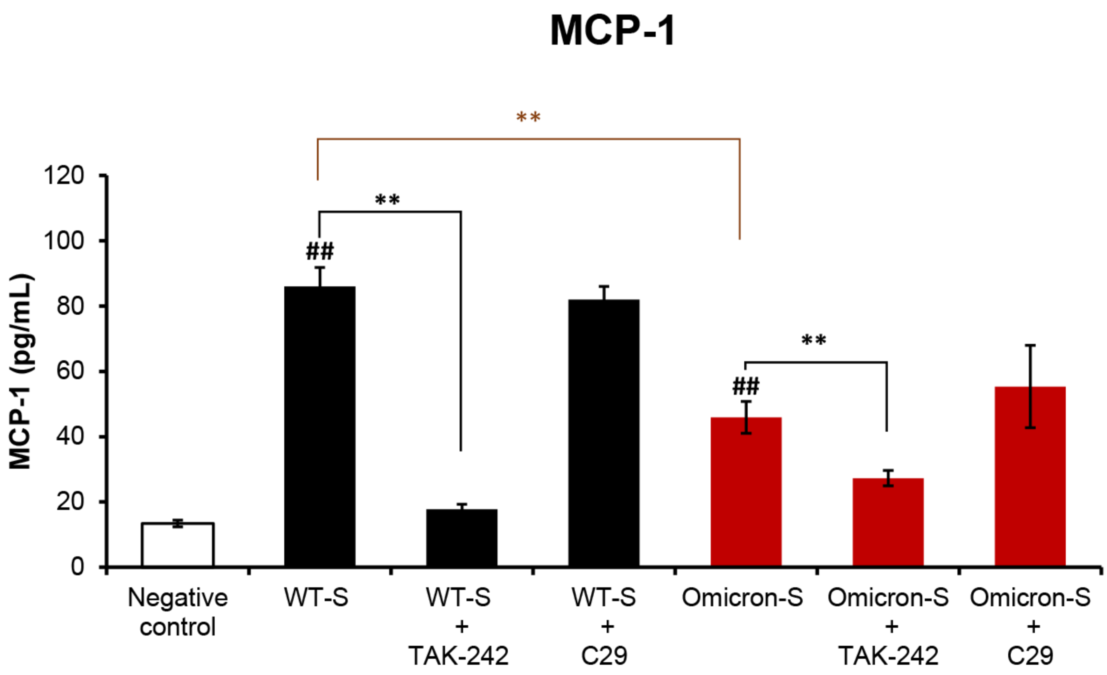

Supplementary Figure S3. Effects of TAK-242 and C29 on supernatant MCP-1 induced by S-protein in THP-1 cells (mean ± SD; n = 3). The cells were treated with WT-S or Omicron-S (7.2 nM) in the presence of TAK-242 (1 μM) or C29 (40 μM). Twenty-four hours later, supernatant MCP-1 was determined using the commercial ELISA kit. The concentrations were calculated from the standard curves. ## *P* < 0.01 *vs*. negative control; ** *P* < 0.01 *vs*. S-protein alone.
